# SOCIAL CAPITAL PROFILES AMONG CHINESE OLDER ADULTS: A LATENT CLASS ANALYSIS

**DOI:** 10.1093/geroni/igae098.3218

**Published:** 2024-12-31

**Authors:** Yuekang Li, Nancy Morrow-Howell

**Affiliations:** University of Chinese Academy of Social Sciences, Beijing, Beijing, China (People’s Republic); Washington University in St. Louis, St. Louis, Missouri, United States

## Abstract

Existing literature often overlooks the multifaceted social engagements of older adults, neglecting how well-being is intricately influenced by diverse combinations of social capital. This study investigates social capital profiles among Chinese older adults, examining how these profiles vary in relation to demographic characteristics and health outcomes. The China Family Panel Study (CFPS) 2016 wave provided data for 7,295 adults aged 60 and above. Based on three levels of social capital (family, community, and societal), Latent Class Analysis (LCA) was employed to identify the social capital profiles of older adults. Multinomial logistic and linear regression models analyze the influence of personal and neighborhood factors on social capital profiles and their correlation with depressive symptoms. Through LCA, three distinct social capital profiles emerged: “Family-centered,” “Family Plus Society,” and “Diverse.” These profiles, each with their unique configurations of social capital, are linked to specific demographic and community features as well as mental health statuses. Notably, this research underscores the particular vulnerability of the “Family-centered” group, characterized by their restricted access to various forms of social capital. Meanwhile, older adults’ social capital is highly connected with the neighborhood they live in. The findings from this study deepen our understanding of social capital by adopting an ecological perspective. The methodology employed to discern social capital compositions and profiles more accurately reflects the diverse social engagements of older adults, offering important insights into the intricate interplay between different levels of social capital and varied older populations.

